# Indications for magnetically controlled growing rods have evolved over time

**DOI:** 10.1007/s43390-025-01262-1

**Published:** 2026-01-21

**Authors:** Katherine D. Sborov, Paal K. Nilssen, Lindsay M. Andras, Michael J. Heffernan, David L. Skaggs, John T. Smith, Paul D. Sponseller, John B. Emans, Peter F. Sturm, Francisco Javier Sánchez Perez Grueso, Kenneth D. Illingworth, Pediatric Spine Study Group

**Affiliations:** 1 Department of Orthopaedic Surgery, Cedars-Sinai Medical Center, 444 S San Vicente Blvd #901, Los Angeles, CA 90048 USA; 2 Jackie and Gene Autry Orthopaedic Center, Children’s Hospital Los Angeles, 4650 Sunset Blvd,Mailstop #69, Los Angeles, CA 90027 USA; 3 Department of Orthopaedics, University of Utah, Salt Lake City, UT 84113 USA; 4 Division of Pediatric Orthopaedics, Johns Hopkins University, Baltimore, MD 21287 USA; 5 Department of Orthopedic Surgery, Boston Children’s Hospital, Harvard Medical School, Boston, MA 02115 USA; 6 Department of Orthopedic Surgery, Cincinnati Children’s Hospital, Cincinnati, OH 45229 USA; 7 Spine Unit, Department of Orthopedic Surgery, Hospital Universitario La Paz, 28046 Madrid, Spain

**Keywords:** MCGR, Scoliosis, Indications, Magnetically controlled growing rods, Early-onset scoliosis

## Abstract

**Purpose:**

The purpose of this study is to determine the trends in use of magnetically controlled growing rods (MCGRs) over time for the treatment of scoliosis.

**Methods:**

All patients treated with MCGRs were identified through a multi-center pediatric spine database from 2014 to 2021. MCGR use over time was analyzed with respect to individual variables within the dataset including demographics, etiology, primary vs conversion surgeries, and major curve magnitude.

**Results:**

A total of 1,404 patients treated with MCGRs were identified. MCGR usage grew quickly until 2017 and then steadily declined through 2021. There was minimal variation over time with respect to patients’ age and weight. MCGR as the index implant as opposed to revision surgery increased from 67% in 2014 to 99% in 2021. Initially, MCGR utilization was consistent across scoliosis etiologies; however, over time, it grew among neuromuscular patients while decreasing among other etiologies. MCGR use decreased over time for curves with magnitude < 60 degrees, with a corresponding rise in curves > 80 degrees.

**Conclusion:**

Use of MCGRs expanded quickly after initial FDA approval in 2014 with broad indications. Over time, the indications for use of MCGRs have steadily evolved. In recent years, a higher proportion of them are used in neuromuscular scoliosis and larger curves in contrast to declining use in smaller curves.

*Level of evidence*: III.

## Introduction

The approval of magnetically controlled growing rods (MCGRs) by the FDA in 2014 marked a significant advancement in the treatment of early-onset scoliosis (EOS) by offering a less invasive alternative to traditional growing rods (TGR) or vertical expandable prosthetic titanium rib (VEPTR) implants [1]. MCGRs utilize magnets to gradually lengthen the rods in the spine externally, allowing growth guidance while eliminating the need for repetitive lengthening surgeries [1]. The appeal of these rods led to rapid expansion of their use; in 2007–2009, MCGRs made up less than 5% of growth-friendly implants but by 2015–2017, 83% of growth-friendly implants were MCGRs [2]. Beginning in 2009, over the course of a decade, there was a 91% decrease in TGR/VEPTR procedures, and a corresponding 479% increase in MCGR insertions [3].

Early studies demonstrated that MCGR was safe and effective for curvature control of EOS, could successfully increase thoracic spine length, and could avoid repeated surgeries for distractions [1, 4, 5]. However, as more patients received MCGR implants and were followed over time, reports emerged of high complication profiles and high rates of unplanned revision surgery [6, 7]. In a review of over 300 MCGR patients, complication rates were reported up to 44% and unplanned revision surgery rate of 33% [8]. Furthermore, the expected psychological benefit of reducing frequent surgeries and hospital visits for rod distraction has been less pronounced than anticipated when controlling for length of follow-up [9, 10]. Considering the complication profile, revision rates, and minimal improvement in quality of life as compared to TGRs, some surgeons are increasingly favoring conservative measures (i.e., bracing, casting, and stalling) until definitive fusion for curves within surgical indications though on the lower spectrum of the major curve magnitude as measured by the Cobb method [11, 12].

Though the use of MCGRs saw a meteoric rise after initial FDA approval, more recently there has been a decline in their use. MCGRs continue to make up a large percentage of implants used in treatment of EOS; however, they are being used in fewer patients over time and their indications for use have likely narrowed. The purpose of this study is to determine the trends in use of MCGR over time for the treatment of scoliosis.

## Methods

In this observational retrospective cohort study, patients were identified via analysis of a multi-center, prospective database from the Pediatric Spine Study Group. All consecutive patients treated with MCGR implants were identified from January 2009, the first recorded use of MCGR, up to December 2021. Patients treated with MCGRs from 2009 to 2013 were ultimately excluded from analysis as there were only 8 total patients treated with MCGR implants in that 4-year period. MCGR gained FDA approval in 2014, after which their use drastically increased.

Data collected from the electronic medical record (EMR) included: demographics, etiology of scoliosis, date of MCGR implant surgery, type of surgery (initial MCGR implant or conversion to MCGR from other treatment modality), and major coronal curve magnitude. Major coronal curve magnitude was measured by the Cobb method by measuring the angle between lines along the superior endplate of the most tilted vertebra at the top of the curve and the inferior endplate of the most tilted vertebra at the bottom of the curve. MCGR use over time from 2014 to 2021 was analyzed with respect to individual variables within the dataset, specifically age at time of MCGR implant, etiology of scoliosis, primary versus conversion surgery, and major curve angles. When analyzing use of MCGR in relation to major curve angles, the major curve was measured on the coronal and/or sagittal view and the largest angle of either the major Cobb angle or the sagittal kyphosis angle was used as reference point.

## Results

**Fig. 1:**
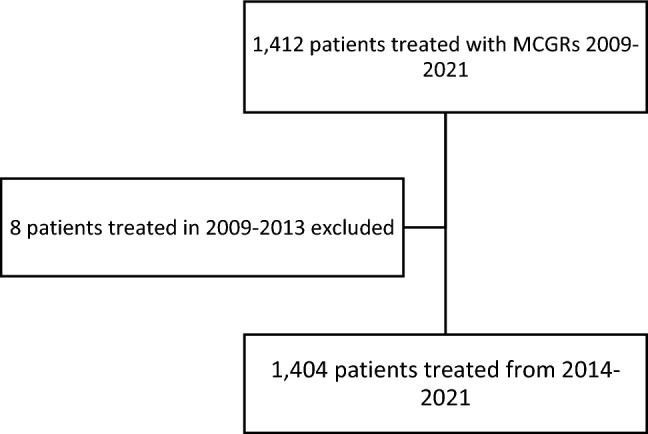
Consort diagram—all patients treated with magnetically controlled growing rods (MCGRs) in the Pediatric Spine Study Group database from 2009 to 2021 were collected

**Fig. 2:**
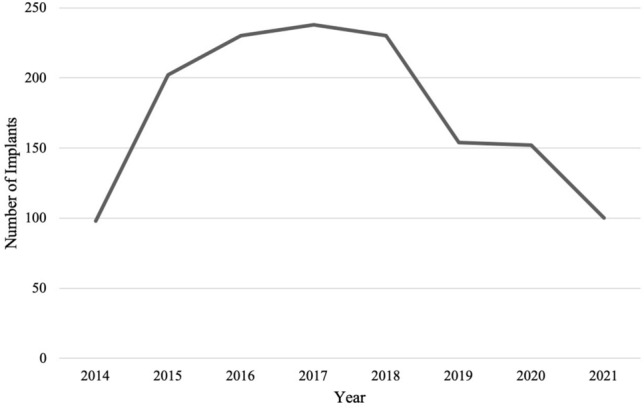
Frequency of magnetically controlled growing rods implants by year

A total of 1,412 patients were treated with MCGRs in the Pediatric Spine Study Group database from 2009 to 2021. After excluding the 8 patients treated in years 2009–2013, there were 1,404 patients between January 2014 and December 2021 (Fig. 1). Overall, the use of MCGRs grew rapidly from their FDA approval in 2014, in which 98 patients were treated, until reaching a peak in 2017, in which 237 patients were treated with MCGR implants. After 2017, their use steadily declined through 2021 where there were only 100 reported patients treated with MCGRs (Fig. 2).

**Fig. 3:**
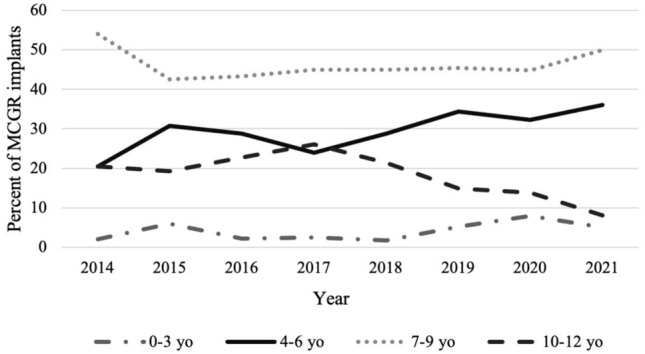
Percent of magnetically controlled growing rods implanted by age group and year

The use of MCGRs over time grew proportionally more in younger patients aged 4–6 years and declined in older patients 10–12 years old. MCGRs were most frequently used in children aged 7–9 years old at all time points studied. In 2014, 20% of implants were used in patients aged 4–6; however, in 2021, the percentage of MCGR use in that age group rose to nearly 40%. Correspondingly, there was a slight drop in patients aged 10–12 from 20% in 2014 to 8% in 2021 (Fig. 3). BMI had no impact on rates of MCGR use or indications over time.

**Fig. 4:**
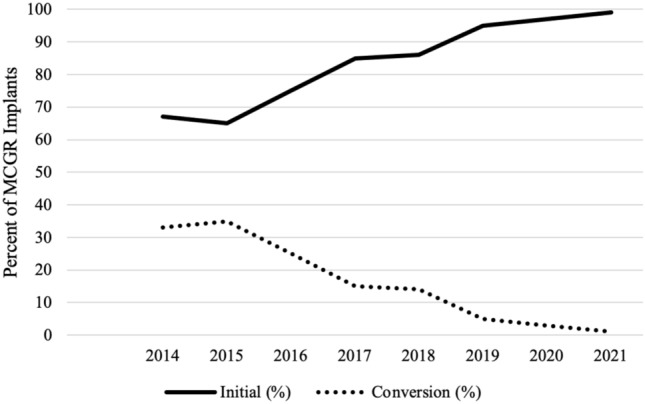
Percent of magnetically controlled growing rods implanted as initial implant versus conversion from prior implant over time

**Fig. 5:**
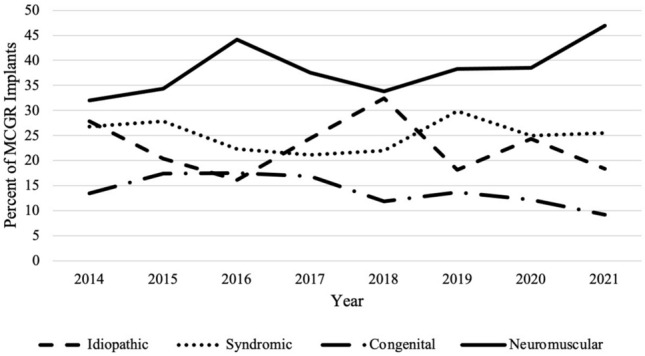
Percent of magnetically controlled growing rods implanted by scoliosis etiology and year

**Fig. 6:**
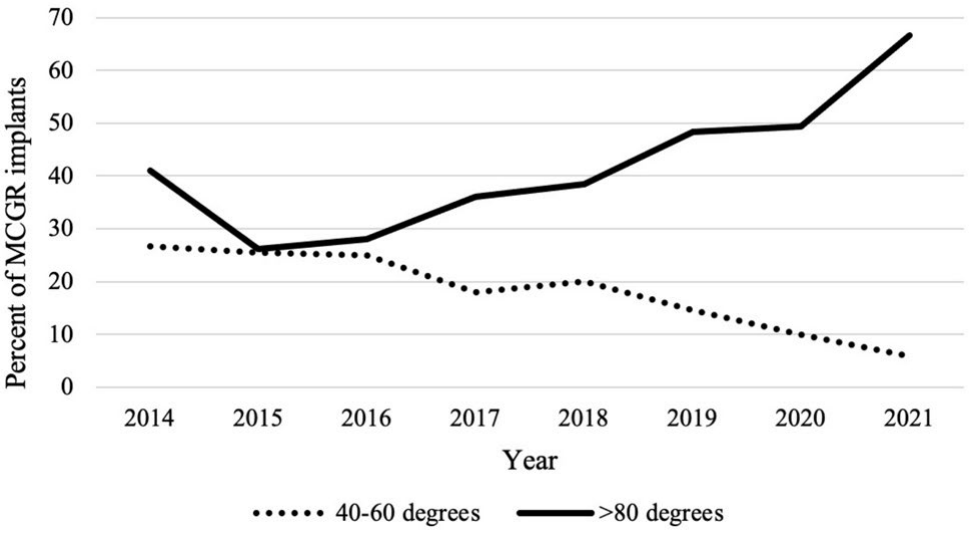
Percent of magnetically controlled growing rods implanted by year and major coronal curve magnitude versus sagittal kyphosis angle

With respect to use of MCGRs as the initial, primary implant versus conversion to MCGR from a different distraction method, e.g., VEPTR or TGR, indications changed drastically over time (Fig. 4). In 2014, 67% of patients were treated with MCGR as their initial implant and 33% of patients were converted to MCGR in a secondary surgery from a different implant. In 2021, 99% of patients were treated with MCGR as initial implant. In early years, the rates of MCGR use in idiopathic, syndromic, neuromuscular, and congenital scoliosis were similar; however, over time, more MCGRs were used in neuromuscular scoliosis with decreasing use in remaining etiologies (Fig. 5). Finally, the use of MCGRs in curves with magnitude < 60 degrees declined slowly over time starting in 2015 with a corresponding steady increase in proportion of patients with severe curves > 80 degrees (Fig. 6).

## Discussion

MCGRs gained rapid popularity after FDA approval, with a peak in 2018; however, in recent years, there has been a narrowing of indications for their use. At their inception, MCGRs were used for almost all patients with EOS with a substantial curve magnitude (e.g., > 40 degrees), observed progression of the curve, and with a large potential for further spine growth [13]. We show that the indications for use have narrowed to predominantly younger patients, though still a high prevalence of use in patients aged 7–9 (Fig. 2). MCGRs are also predominantly used in neuromuscular EOS and large curve magnitudes > 80 degrees. Our findings of the rise and fall of MCGR usage mirror two prior studies characterizing use of growth-friendly implants for EOS over time that demonstrate an overwhelming shift toward the use of MCGR by 2017–2018 with a corresponding declining use of VEPTR and TGR followed by a steady significant decline in use of MCGR from 2018 to 2019 and again from 2019 to 2020 [2, 3]. While pediatric spine journals and meetings saw a rapid growth in MCGR-related research during their rise in popularity [14], this is the first study to examine the narrowing indications of MCGRs in recent years.

A great hope of MCGRs was the psychosocial impact on children and families by saving them from the need for repeated surgical procedures and hospitalizations from TGRs. Flynn et al. [15] and Aslan et al. [16] assessed the psychological well-being of children with EOS treated with TGRs and found a high prevalence of depression and anxiety. An intended benefit of the MCGR system was to minimize this burden. In recent studies of health-related quality of life (HRQOL) outcomes at least 2 years after surgery, patients treated with MCGR had more improvement in quality of life, as measured by the Early Onset Scoliosis Questionnaire (EOSQ), compared to patients treated with TGRs, but findings did not reach statistical significance [9–11]. Notably, studies investigating long-term effects of MCGRs on HRQOL and EOSQ are limited by sample sizes and are consequently underpowered. Further research is necessary to better understand the burden of care and psychological impact on children with EOS.

Initials studies of patients treated with MCGR showed promise in correcting spine curvature and promoting growth of the vertebral column without the burden of repeated surgical procedures [5, 17, 18]; however, subsequent studies reported high complication rates and need for revision surgeries [6–8]. Long-term follow-up studies revealed complications rates similar to TGRs [19, 20], with some studies reporting rates as high as 44% [8]. Commonly reported complications included pullout of proximal anchors, loss of distraction mechanism, rod breakage, and tissue necrosis [8, 21]. Implant-related complications are due in part to the design of the implant itself but may also be related to surgical technique and surgical decision making, particularly with regard to implant size and rod contouring [8, 22]. Despite etiology of the complication, the rate of unplanned operations at 2 years post-implant is high, ranging from 23 to 50% [6–8, 13, 23]. Common causes included revision of fixation, premature rod exchange, early definitive fusion, and addressing other complications such as infection and tissue necrosis [7, 8, 13]. Thus, MCGRs have not completely avoided repeated invasive surgical procedures as previously hoped.

As compared to conventional rods, MCGRs have high initial costs due to the expense of the implants; however, they reach cost equivalency with traditional rods about 4 years from the initial implant date [24–26]. Further, the law of diminishing returns demonstrated in TGRs [27], or the concept that with repeated lengthening’s the magnitude of gain is diminished, has been repeatedly demonstrated in MCGRs [28–30]. At about 2.5 years after implantation, only around 25% of the intended distraction is achieved [27]. The challenges encountered by MCGRs (i.e., complication profile, unplanned operation rate, cost, and equivocal improvement in quality of life) may have all contributed to the change in indications over time.

In recent years, surgeons have had increasing preference for conservative management with bracing and casting moderate curves, delaying surgical intervention [2, 11, 12, 31]. In this study, we see decreasing use of MCGRs in moderate curves, < 60 degrees, and increasing use for more significant curves > 80 degrees. An evolving paradigm shift to more conservative “stalling” treatment options, such as bracing and serial casting or proceeding directly to definitive fusion and avoiding growth-friendly surgery all together likely contributed to these findings in smaller curve magnitudes [2, 3, 12, 31]. These delay and “stall” tactics likely aim to decrease the complications associated with growth-friendly surgery with a “one and done” approach.

There are a number of limitations in this retrospective, observational study. Most notable limitations include the COVID-19 pandemic in 2020 and the MCGR recall in 2020. The COVID-19 pandemic impacted overall number of surgeries performed across the board and certainly impacted the number of EOS patients treated operatively. At a similar time, NuVasive issued a voluntary recall of their MACEC X rods due to a 0.5% risk of post-implantation separation of an actuator end-cap component [32]. Finally, though MCGRs have been reportedly used less frequently from 2018 to 2021, they are the predominant implant used in surgical treatment of EOS. As this is a registry study, the study is limited to only data that have been entered by participating sites within the PSSG and is not a full reporting of all MCGR usage. Furthermore, the study is dependent on accurate reporting to the PSSG by participating sites; perhaps there has been a decrease in reporting of MCGR use by participating sites. These limitations aside, the data studied to determine trends in indications for MCGR use over time were studied as a proportion of total cases and should not be impacted by the pandemic, recall, nor overall reporting. Finally, inherent to the nature of this purely observational study, we are unable to determine statistical significance of the observed trends.

## Conclusion

In this study of all patients treated with MCGR implants since their FDA approval in 2014, we show a rapid rise in popularity of MCGRs until reaching a peak in 2017, followed by a decline in their overall use starting in 2018. While indications for MCGR use were broad initially, indications for their use have narrowed. As overall use of MCGRs decrease, a higher proportion of them are used in younger patients, neuromuscular scoliosis, and larger curves in contrast to declining use in older patients and in smaller curves.

## Funding source

No external funding was secured for this study.

## Data Availability

The data that support the findings of this study are not publicly available, but are available from the corresponding author upon reasonable request.
